# Evaluation of Activity of Sesquiterpene Lactones and Chicory Extracts as Acetylcholinesterase Inhibitors Assayed in Calorimetric and Docking Simulation Studies

**DOI:** 10.3390/nu14173633

**Published:** 2022-09-02

**Authors:** Andrzej Jaśkiewicz, Grażyna Budryn, Miguel Carmena-Bargueño, Horacio Pérez-Sánchez

**Affiliations:** 1 Faculty of Biotechnology and Food Sciences, Institute of Food Technology and Analysis, Lodz University of Technology, 90-537 Lodz, Poland; 2 Structural Bioinformatics and High-Performance Computing Research Group (BIO-HPC), Computer Science Department, Catholic University of Murcia (UCAM), Guadalupe, 30107 Murcia, Spain

**Keywords:** chicory, sesquiterpene lactones, acetylcholinesterase, isothermal titration calorimetry, molecular docking simulation, Alzheimer’s disease

## Abstract

The aim of the study was to explain the effects of sesquiterpene lactones (SLs) from chicory (*Cichorium intybus* L.) root extracts as inhibitors of acetylcholinesterase (AChE) at the molecular level and to determine the inhibition of AChE activity by specific SLs (lactucin and lactucopicrin) and different chicory extracts. The obtained SLs-rich extracts were purified by the countercurrent partition chromatography (CPC) technique. AChE inhibitors were analyzed using two models: isothermal titration calorimetry (ITC) and docking simulation. The results of ITC analysis of the enzyme and the ligands’ complexation showed strong interactions of SLs as well as extracts from chicory with AChE. In a test of enzyme activity inhibition after introducing acetylcholine into the model system with SL, a stronger ability to inhibit the hydrolysis of the neurotransmitter was observed for lactucopicrin, which is one of the dominant SLs in chicory. The inhibition of enzyme activity was more efficient in the case of extracts, containing different enzyme ligands, exhibiting complementary patterns of binding the AChE active site. The study showed the high potential of using chicory to decrease the symptoms of Alzheimer’s disease.

## 1. Introduction

An important kind of disorders affecting modern society, mainly the elderly, are neurodegenerative diseases. Neurodegenerations, including Alzheimer’s disease (AD), are incurable and lead to the gradual degeneration and death of nerve cells. This results in problems with movement (ataxia) or a decline in mental performance (dementia), and eventually, death.

Due to the direct relationship between the acetylcholine (ACh), a parasympathetic neurotransmitter, deficit and the severity of dementia, AD is preferentially treated with acetylcholinesterase (AChE) inhibitors. AChE belongs to the group of hydrolases acting on the ester bond of carboxylic acid esters (3.1.1.7) [1]. AChE is involved in the breakdown of ACh into choline and acetate, which are re-taken up for de novo neurotransmitter synthesis, and inhibition of AChE increases the concentration of ACh in the postsynaptic cleft, leading to an exacerbation of the cholinergic response over nerve structures requiring parasympathetic stimulation, such as neuromuscular junctions [2]. Nowadays, available drugs inhibiting human AChE include substances such as rivastigmine donepezil or galantamine, that, with long-term use, often show adverse side effects. The most common complications associated with taking these inhibitors include heart problems, severe gastrointestinal and liver disorders, diarrhea, insomnia, fainting, nausea and vomiting, muscle cramps, fatigue, headache, dizziness and weight loss. As an answer to this problem, natural, milder AChE inhibitors are sought, mainly from plant sources. Many studies have confirmed the beneficial activity of AChE inhibition by plant extracts. An example is an extract from sweet pepper, which contains flavonoids, phenolic acids, and carotenoids [3]. Some reports suggest that alkaloids, such as uleine from *Himatanthus lancifolius,* could be considered as AChE inhibitors [4]. Although, in the case of coffee containing the alkaloid caffeine and phenolic acids, the latter were mainly shown to inhibit the hydrolysis of ACh. [5]. The review by dos Santos at al. [6] compared a number of natural extracts that exhibit AChE inhibitory activity. The substances found in the most active extracts included: decursinol, berberine, palmatine, groenlandicine, ateorrhizine, mesuagenin, serpentine and coptisine. It was also confirmed that terpenes and phenylpropanoids can act as cholinesterase inhibitors. Arya et al. [7], in the review article on non-alkaloid AChE inhibitors, listed sesquiterpene lactones as an important group of potential therapeutics, including: amberboin, lidiol, sibthorpine, gaillardine, amberine and 7-hydroxyfrullanolide. 

The reach source of terpenes from the group of sesquiterpene lactones is common chicory (*Cichorium intybus* L.), a vegetable belonging to the *Asteraceae* family. The leaves and flowers of the plant are usually used as ingredients of salads and the roots are processed to obtain a soluble dietary fiber preparations [8,9]. Some studies have reported on the pro-healthy impact of *Cichorium intybus* extracts, including neuroprotective activity [10]. Recent studies have shown that sesquiterpene lactones (SLs) can be responsible for these effects [11,12]. Lactucin, lactucopicrin and their derivatives, which are chicory-specific SLs, are physiologically active in many areas including antidiabetic, anti-inflammatory, antioxidant, anticancer and moderate antinociceptive activities [13,14,15].

The purpose of the study was to assess the molecular effect of SLs as AChE inhibitors and to determine the inhibition of AChE activity by specific SLs (lactucin and lactucopicrin) and chicory extracts, depending on conditions of obtaining and purification. 

## 2. Materials and Methods

### 2.1. Materials, Chemicals and Reagents

LC-MS-grade reagents water (≥95%), methanol (≥99%), formic acid (≥98%), acetylcholine chloride (≥99%) and acetylcholinesterase (EC 3.1.1.7 from electric eel, lyophilized powder 200–1000 U/mg) were purchased from Sigma-Aldrich (St. Louis, MO, USA), *n*-hexane (≥99%) and ethyl acetate (≥99%) from Chempur (Piekary Śląskie, Poland), and lactucin (≥95%) and lactucopicrin (≥95%) from Extrasynthese (Genay CEDEX, France). Nylon syringe filters were purchased from Chromacol (Herts, UK). Ultrapure distilled water (18.2 MΩ cm) was obtained on a Millipore Milli-Q Plus system (Bedford, MA, USA). Fresh chicory roots (*Cichorium intybus* L.) were obtained from a local chicory breeder Bakor cultivating the vegetable using the hydroponic method (Skierniewice, Poland). 

### 2.2. Preparation of Chicory Root Extracts

The extracts with the use of a pressure vessel were prepared in accordance with the method proposed in the previous study [16].

Briefly fresh chicory roots after cleaning and grinding (1300 g) were mixed with the solvent in the amount of 2100 mL and extracted with the method proposed by Budryn et al. [17] with some modifications, in a pressure vessel type PS-5692 (Vienna, Austria). As a solvent, a water–methanol mixture (70/30 or 50/50, *v*/*v*) was used at 80 °C for 20 min. The pressure in the vessel during extraction was 0.2 MPa. The obtained suspensions were filtered using the KNF 18 035.3 N vacuum pump (Neuberger, NJ, USA) and paper filters with a density of 84 g/m^2^ Poch (Gliwice, Poland). The methanol was then evaporated at 446 mbar in a Rotavapor R-210/215 evaporator (Büchi, Switzerland). The obtained extracts, devoid of organic solvent, were frozen at −80 °C for 24 h and freeze-dried (Delta 1-24 LSC lyophilizer, Martin Christ GmbH, Osterode am Harz, Germany). The obtained lyophilized preparations were stored at −25 °C before further analysis.

### 2.3. Purification of Chicory Extracts

Freeze-dried extracts were purified using countercurrent partition chromatography (CPC) on a SPOT Prep II 50 chromatography apparatus (Armen Instrument, Saint-Avé, France) integrated with a UV/VIS detector and fraction collector. For purification, a modified method proposed by Destandau et al. [18] and Wu et al. [19] was adopted. The CPC technique is used to separate the components of mixtures in a two-phase liquid-liquid system, without a fixed bed. The purification of chicory extracts was carried out to decrease the concentration of inulin. In order to separate sesquiterpene lactones (SLs) from other components, a two-phase system of solvents was prepared after preliminary studies, which consisted of, respectively, lower and upper, less and more hydrophobic phases.

A two-phase solvent system was prepared from *n*-hexane, ethyl acetate, methanol, and water in the ratio of 7:3:5:5 (*v*/*v*/*v*/*v*). The solvents were placed in the separating funnel, mixed and partitioned into two phases. The phases obtained were used as mobile (upper) and stationary (lower) phase, respectively. They were passed through each other in ascending mode in the CPC apparatus. The technique used takes advantage of the differences in the values of the partition coefficients of the purified components between the two phases in a given solvent system, contributing to the elution of the analytes at different times.

**Figure 1:**
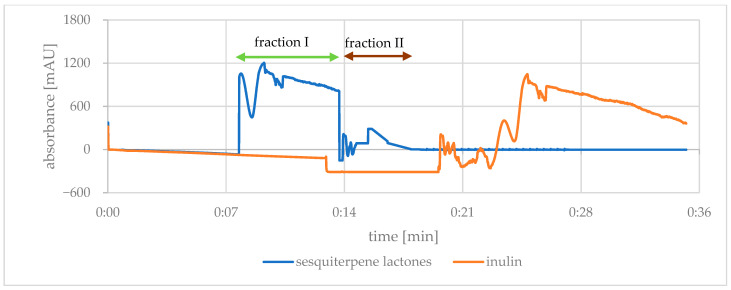
CPC (countercurrent partition chromatography) chromatogram of the separation of *Cichorium intybus* extract, sample 2 g/25 mL of solvent system: n-hexane:ethyl acetate:methanol:water (7:3:5:5, *v*/*v*/*v*/*v*); flow rate: 30 mL/min, rotation speed: 500 rpm; detection wavelength: 254 nm (sesquiterpene lactones), 285 nm (inulin).

Separation of the components of chicory extracts was made by filling the 250 mL CPC rotor with the lower phase. The rotor speed in the filling step was 1400 rpm and the flow rate was 8 mL/min, within 15 min. Then, in the purification step, a 2 g sample of the lyophilized extract (50/50 or 70/30) was dissolved in 25 mL of the lower phase and filtered through a nylon syringe filter (0.45 µm). The filtrate was injected into the rotor. In the next step, the upper phase was passed through the rotor. Parameters used: flow rate 30 mL/min and rotational speed 500 rpm. The flow of this phase lasted 20 min, during which the components were eluted, starting with the most hydrophilic. Then, after elution, the extrusion of the rotor content with the lower phase was started, during which the stationary phase, containing the remaining analytes, was gradually removed, enabling further chromatographic separation of the components. The extrusion phase ran for 15 min. The composition of the eluted mixture was monitored by detecting the absorbance of the eluate at 254 nm, the maximum absorbance of the SLs. The obtained chromatogram (Figure 1) was analyzed with the Armen Glider CPC v5.0b.11 software. The elution of SLs from the chicory extracts occurred between 8 and 13 min (more hydrophilic fraction assigned as I) and between 14 and 18 min (more hydrophobic fraction II) of the analysis, recorded as high UV absorbance at 254 nm. The eluate was collected in the collector as fractions in 25 mL glass tubes and the contents of tubes were combined according to the course of the curve to give fractions I and II. The obtained fractions, after removal of the organic solvents, were frozen and freeze-dried as above. The freeze-dried purified extracts were stored at −25 °C until analysis.

### 2.4. Analysis of Sesquiterpene Lactones Concentration

The concentration of sesquiterpene lactones (SLs) in chicory root extracts was determined using ultra-high-performance liquid chromatography with electrospray ionization and mass spectrometry (UHPLC-ESI-MS) using the modified method of D’Antuono et al. [20] and in accordance with Jaśkiewicz et al. [16]. The extracts were dissolved in an ultrapure water:methanol mixture in a proportion corresponding to the composition used for the initial extraction, 70/30 or 50/50, *v*/*v*, (20 mg/mL) and filtered using a nylon syringe filter (0.2 µm). Chromatographic analysis was carried out using the mass spectrometer LCMS-2020 (Shimadzu, Tokyo, Japan) with electrospray ionization source. The chromatographic separation was performed using an Accucore-150-C18 column (150 mm × 3.0 mm × 2.6 µm; Thermo Scientific, Waltham, MA, USA) thermostated at 30 °C. The extract solutions were placed in an autosampler; 4 µL of the sample was injected onto the chromatographic column and a gradient elution was performed. Mobile phase A consisted of water and formic acid (99.9:0.1 *v*/*v*), and mobile phase B from methanol and formic acid (99.9:0.1 *v*/*v*). A flow rate of 0.2 mL/min was used. The gradient elution was as follows: 0–20 min at 100–58% A, 20–30 min at 58% A, 30–45 min at 58–0% A, and 45–50 min at 0% A. Calibration curves were constructed for standard substances (lactucin and lactucopicrin) using 6 concentrations in the range of 0.01–1.0 mg/mL. MS spectra were acquired in collision-induced dissociation (CID) mode using nitrogen. The mass spectrometric conditions were as follows: capillary voltage 4500 V, drying-gas temperature 250 °C, drying-gas flow 15.0 L/min, and capillary temperature 350 °C, and nitrogen was used as the nebulizer. Full-scan mass spectra were acquired over the *m*/*z* mass range from 50 to 2000 in a negative ion mode. SLs identification was based on a comparison of the recorded MS spectra with that of standards analyzed under identical conditions, characterized by *m*/*z* for lactucin and lactucopicrin. The remaining SLs were identified by specific MS spectra [21,22]. LabSolutions 5.60 was used to control the apparatus, data collection and calculations. 

### 2.5. Isothermal Titration Calorimetry (ITC)

The evaluation of the activity of the obtained chicory extracts as inhibitors of acetylcholinesterase (AChE) was performed by calorimetric measurements using the isothermal titration calorimetry (ITC) method with the use of the MicroCalPEAQ-ITC200 calorimeter (Malvern, Worcestershire, UK). The analyses were performed according to Budryn et al. [23] with some modifications. A 0.2 mL calorimetric cell was filled with a degassed 1 μmol/L solution of the enzyme dissolved in water. A water:methanol solution with a concentration of 1 mmol/L (converted to lactucin content) of purified extracts (70/30 and 50/50) was injected into the vessel, where the analytes were diluted in the enzyme solution and simultaneously were bound to AChE. The concentration of methanol in the vessel after titration did not exceed 7%. The analysis was performed at 36.6 °C with continuous stirring (307 rpm). The extracts were injected at intervals depending on the duration of the observed thermal effects, which should return to equilibrium after each injection. The analysis was also performed with the standards of the two SLs from chicory: lactucin and lactucopicrin, at a concentration of 1 mmol/L. instead of extracts’ solutions. The chosen SLs significantly differed in the structure, possessing OH or O_2_CCH_2_PhOH moiety, respectively, which may have resulted in a different nature of interactions with the enzyme. The heat released by the interaction of AChE and the inhibitors (the extracts or the single substances) was recorded over time and raw data were obtained as a graph of heat flow in μcal/s versus time. Integration of each peak gave the value of the heat released during the injection of the inhibitor solution into the cell filled with AChE. Plots of heat of interactions versus molar inhibitor:AChE ratio were then used to determine the thermodynamic parameters of the interactions such as: dissociation constant (K_D_), binding constant (K_A_), enthalpy change (ΔH) and entropy change (ΔS), which were calculated using the least squares nonlinear saturation curve-fitting method performed with the MicroCal PEAQ-ITC200 software. The “single binding site” mode was used. The free energy change (ΔG) was calculated from the Gibbs Equation (1) [24,25].

∆G = ∆H − T∆S(1)

The Michaelis constant (Km) was obtained by calculating the fit of the Michaelis–Menten equation with the saturation curve obtained by titration of AChE (1 μmol/L) with acetylcholine (Ach) (1 mmol/L) using nonlinear regression and MicroCal PEAQ-ITC200 software. The addition of inhibitor (SLs or extracts at 1 mmol/L) to the enzymatic reaction of ACh with AChE limited the hydrolysis of the neurotransmitter. The difference in the heat of acetylcholine hydrolysis in the presence of the inhibitors (SLs or extracts) (ΔH_AChE-ACh-H-I_) and without inhibitors (ΔH_AChE-ACh-H_) was used to calculate the inhibitory activity (IA) (2) and the IC50 concentration of the inhibitor (converted to lactucine in the case of extracts), which caused a 50% decrease in the enzyme activity. The heat of acetylcholine hydrolysis was corrected for heat values: the signal of the interaction between AChE and the SLs or extracts in case of the inhibition test (ΔH AChE–inhibitor interactions, ΔH_AChE-I-I_) and for the signal of injection of the ligand (ACh and SLs/extracts) into the cell without the enzyme (ΔH ACh/inhibitor dilution, ΔH_ACh-D_, ΔH_I-D_).

IA% = [(ΔH_AChE-ACh-H_ − ΔH_ACh-D_) − (ΔH_AChE-ACh-H-I_ − ΔH_AChE-I-I_ − ΔH_ACh-D_ − ΔH_I-D_)] × 100/ΔH_AChE-ACh-H_ − ΔH_ACh-D_ (2)

Using the same procedure as for Km, the inhibition constant (Ki) was calculated, which was the dissociation constant of the enzyme–inhibitor complex. 

### 2.6. Molecular Modeling

Molecular modeling simulated the docking of sesquiterpene lactones to acetylcholinesterase (AChE). The research, with the use of molecular modeling, allowed us to obtain detailed information at the atomic level on the interaction type (van der Waals interactions, hydrogen binding and hydrophobic interactions) between SLs from chicory extracts and the enzyme, responsible for the inhibitory effects. For this purpose, representative AChE X-ray structure (1EVE) was taken from the Protein Data Bank database. In the next step using the Protein Preparation Wizard tool, that is available in the Maestro software [26], appropriate enzyme models were prepared; the hierarchy of bonds was established and hydrogen atoms were added. In this tool, we selected the following options: “Assign bond orders”, “Use CCD database”, “Add hydrogens”, “Create zero-order bonds to metals”, “Create disulfide bonds” and “Generate heat states using Epik pH 7 ± 2.0” [27]. In the next step, using the tool System Builder, the charges were assigned based on ForceField OPLS3e [28]. The chemical structures of the lactucin and lactucopicrin molecules were built and fully optimized using the tool of Maestro Lig-Prep. This tool uses Epik [27] to calculate the protonation state of each molecule (the pH used was 7 ± 0.5) and it assigned the charges of each atom with the ForceField OPLS3e [28]. 

Docking of chicory sesquiterpene lactones to the prepared enzyme model was performed with the Lead Finder docking program [29] using default parameters. The size of the ligand-docking mesh was set as 30 Å in each direction from the geometric center for each individual docking simulation. The evaluation function of the Lead Finder program takes into account the Lennard–Jones factor, hydrogen bonds, electrostatic interactions, stabilizing hydrophobic interactions and correction of entropy for the number of binding turns and the ligand internal energy. The representation of each interaction was performed using Poseview software [30]. All these calculations were run using metascreener (https://github.com/bio-hpc/metascreener, accessed on 3 February 2022)).

### 2.7. Statistical Analysis

Extraction was performed independently in triplicate and analyses of each compound, extract or fraction were performed in triplicate. Statistical analysis was performed with the use of Statistica 13.1 software. In order to evaluate the normal distribution of groups, the Shapiro–Wilk test was performed. Additionally, Levine’s test was performed to confirm the homogeneity of variance, followed by one-way analysis of variance (ANOVA) to compare the results and Tukey’s test to reveal pairs of groups that differed from statistical significance in terms of means. Significance was defined at *p* ≤ 0.05.

## 3. Results and Discussion

### 3.1. Chemical Composition of Chicory Root Extracts

Table 1 summarizes the results of the sesquiterpene lactones (SLs) concentrations in raw and purified extracts obtained from chicory roots. The sum of SLs in the raw extracts prepared using a pressure vessel was 3.457 and 4.479 g/100 g db., depending on the solvent used. The total SLs in the obtained extracts increased by about 200% as compared to the extraction under atmospheric pressure which was used in the previous study [16]. This could be the effect of cell walls’ increased permeability under the influence of increased pressure. Rivera-Tovar et al. [31] demonstrated that increased temperature and pressure enhanced both the mass transfer and solubility of bioactive compounds from plant material, as well as reduced solvent viscosity. It was observed that a lower concentration of methanol in the solvent was more beneficial for the extraction of sesquiterpene lactones. Willeman et al. [21] postulated that adding water to methanol during the extraction of SLs from chicory root broadened the polarity range and improved extractability. Water reduced the dehydrating effect of MeOH and promoted its diffusion in the matrix, allowing better penetration of the solvent mixture, dissolved target compounds more widely and increased extraction. Under atmospheric pressure and ambient temperature, the swelling of the membranes caused by water addition was the limitation; it promoted the penetration of alcohol, and the water content was optimal at 25% [21]. The results presented in Table 1 show that the increase in both temperature and pressure during chicory root extraction overcame the resistance of the swollen material of the cell walls and allowed good solvent penetration even with a proportion of water to methanol of 75:25 (*v*/*v*), making it possible to fully utilize a wide range of polarity of the solvent mixture. In the case of the purified extracts, the total SLs content ranged from 2.276 to 4.278 g/100 g of db. The highest concentration of total SLs was characterized by more hydrophobic fraction CPC 70/30 II, and the lowest by CPC 50/50 I.

Using the CPC (countercurrent partition chromatography) technique allowed obtaining new preparations in terms of SLs composition, as in the case of the study by Wu et al. [19] with the extract of *Cichorium glandulosum* root obtained with ethyl acetate applying the method of flash countercurrent chromatography (HSCCC). The individual SLs differ in both the partition coefficient (logP) between the upper and lower phases of the solvent mixture as well as in the polarity of the surfaces. The tested SLs were characterized by low lipophilicity [31], yet they were mostly eluted during the CPC analysis in fraction I with a less hydrophobic solvent system. Fraction II, eluted with a more hydrophobic solvent system, revealed residues and significantly smaller amounts of SLs (approx. 3% of fraction I, Figure 1), while inulin was eluted only during the extrusion phase. The concentration of 8-deoxylactucin and 8-deoxylactucin oxalate was higher in fractions CPC II, while of lactucin, 11(S),13-dihydrolactucin, lactucopicrin and 11(Z),13-dihydrolactucopicrin, the concentration was higher in fractions CPC I.

### 3.2. Evaluation of Sesquiterpene Lactones Preparations as AChE Inhibitors 

The purified preparations of chicory extracts were devoid of most of the inulin and contained a relatively high concentration of SLs, which showed in other studies a wide spectrum of pro-health properties, including those affecting the nervous system. An important role in the conservative therapy of Alzheimer’s disease is played by drugs from the group of acetylcholinesterase inhibitors (AChE), which limit the progression of the disease and its acute symptoms. Synthetic drugs with an AChE-inhibiting effect have serious side effects after long-term use, and, therefore, harmless inhibitors of natural origin with a similar mechanism of action on cholinesterases are sought [32,33,34]. In the study, to assess the activity of chicory extracts as AChE inhibitors, isothermal titration calorimetry (ITC) was used. Only purified extracts containing residual amounts of inulin, undesirable during calorimetric titration, were analyzed by ITC. The method allows obtaining information on the interactions of the enzyme with the potential inhibitor and also on limiting the enzymatic hydrolysis [5].

**Figure 2:**
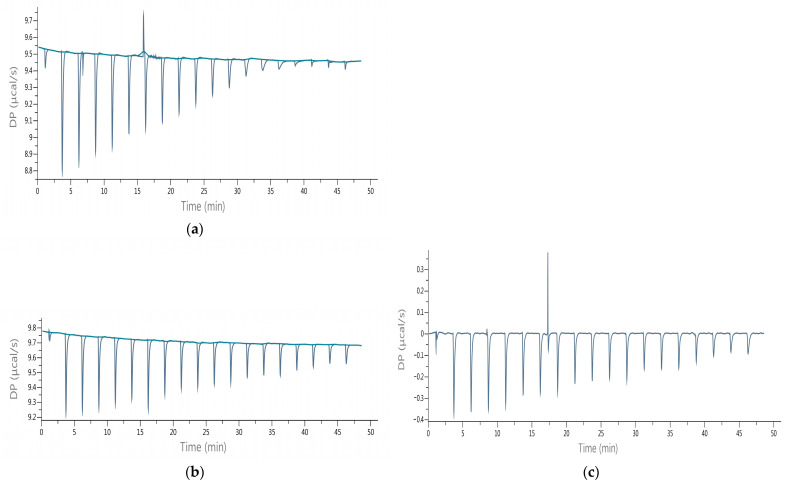

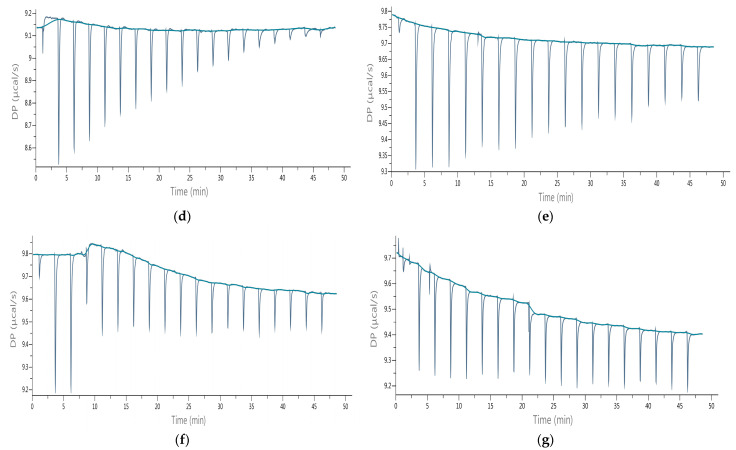
ITC raw data of titration of acetylcholinesterase (AChE) at a concentration of 1 μmol/L 1 with ligands (acetylcholine (Ach), sesquiterpene lactones (SLs) or purified chicory extracts by countercurrent partition chromatography (CPC) at concentrations of 1 mmol/L (converted to lactucin content for extracts); (**a**) hydrolysis of ACh by AChE; reduced hydrolysis of ACh by AChE with: (**b**) lactucin; (**c**) lactucopicrin; (**d**) CPC 70/30 I extract; (**e**) CPC 70/30 II extract; (**f**) CPC 50/50 I extract; (**g**) CPC 50/50 II extract.

The experiments were performed with standard substances (lactucin and lactucopicrin), and then with the obtained purified extracts, which allowed evaluating the activity of the preparations in comparison to the pure SLs. The thermodynamic parameters of interactions were determined, such as: ΔH, ΔG, ΔS, K_D_, and K_A_. In a separate experiment, [31the influence of the formed complexes on the course of the hydrolysis of the physiological AChE substrate, i.e., Ach was evaluated and values of IA%, IC_50,_ and Ki were calculated. Table 2 summarizes the obtained thermodynamic parameters of the interactions determined on the base of the raw data given in Figure 2.

The change in enthalpy (ΔH) of the interactions of the formed AChE–inhibitor complexes was negative indicating the exothermic nature of the binding. During ACh hydrolysis without inhibitors and in the presence of lactucopicrin, a single endothermic peak occurred during the titration (Figure 2) which could demonstrate the conformational changes in the enzyme caused by atomic forces of bound molecules. The total energetic effects ranged from −166.93 to −57.72 kJ/mol. The interactions of lactucin with the enzyme were characterized by the lowest negative enthalpy change compared with lactucopicrin, and the interactions of the purified extracts exhibited aligned ΔH values except of CPC 70/30 I which was characterized by the lowest negative enthalpy change.

ITC thermodynamic analysis allowed the determination of the affinity (ΔG) of AChE for the tested inhibitors. The ΔG values of the analyzed pairs were on a similar level but some trends could be observed. CPC 70/30 I preparation showed the lowest negative value of ΔG = −20.94 kJ/mol, while the highest (−23.41 kJ/mol) was characteristic for lactucin, comparable to that of CPC 50/50 II (*p* < 0.05). The high negative values of ΔG suggest van der Waals interactions and hydrogen bond formation. The change in entropy ΔS of the performed interactions was within the range from −289.61 to −212.14 J/mol × K. The highest negative change in entropy was obtained in the case of CPC 50/50 I, while the lowest was for CPC 70/30 I. A negative ΔS indicates non-covalent interactions that can be stabilized by hydrogen bonds [35]. The dissociation constant (K_D_) of the complexes ranged from 29.90 to 299.02 µmol/L, indicating the complex with lactucin as the most stable. The binding constant (K_A_) of the interactions of lactucopicrin and lactucin amounted to 5.92 and 33.44 × 10^3^ L/mol, respectively (Table 2). The K_A_ depended on the number and energy of different types of interactions which were further determined in detail by docking simulation.

The K_D_ determined for extracts was higher than that of the lactucin and lactucopicrin. Nevertheless, they quite effectively inhibited the enzyme, probably as a result of binding more than one ligand at different sides by interactions with a number of amino acid residues, that could be confirmed by docking simulation. The calculated AChE-inhibitory activity (IA) of the tested substances and extracts ranged from 26.23 to 98.61%. The lowest activity was shown by lactucin and about three times higher was for lactucopicrin. The purified extracts showed activity as AChE inhibitors near 100% at the tested concentrations, except for CPC 70/30 I, for which the activity was close to 90%. The IC_50_ concentrations of the ligands were inversely proportional to the IA and ranged from 1.51 (CPC 50/50 I) to 3.11 μmol/L (lactucin). The activity of the purified extracts as AChE inhibitors was similar, regardless of the SLs profile. No significant correlation was found between the concentration of the individual SLs and the inhibitory activity of the enzyme, showing that a mixture of SLs is much more active than a single compound despite the same SLs molar concentration of purified extracts and standards. 

The Michaelis constant K_m_ of ACh hydrolysis by AChE amounted to 48.50 µmol/L, which was lower than that determined by Xu et al. [36] using quantitative matrix-assisted laser desorption/ionization Fourier-transform mass spectrometry. The obtained inhibition constant (K_I_) values of the tested substances and extract were in a narrow range from 1.12 (lactucin) to 1.43 µmol/L (CPC 50/50 I). 

The serum concentration of free lactucin and 11(S),13-dihydrolactucin after consumption of a serving of chicory juice containing 49 μmol of total SLs showed a Cmax of 0.143 and 0.116 µmol/L, respectively, and the Cmax of total SLs amounted to 0.284 µmol/L determined by Weng et al. [37]. It suggests the in vivo efficacy of the tested extracts containing even about 200 μmol of total SLs converted to lactucin in a dosage of 2 g of extracts with approx. 3% of SLs, four times higher than in the juice. As a consequence, consumption of about 2 g of extracts could elevate the Cmax for total SLs, even to a level close to the determined inhibitory constants, although the potential effect must be proven in clinical trials as many physiological factors such as type of transport, saturation of receptors, efflux, crossing the intestinal and the blood–brain barriers, etc., may influence the actual plasma concentration of the discussed inhibitors. 

According to the relation that Ki equaling the maximum plasma drug concentrations makes the drug likely to inhibit the activity of the enzyme, the useful preparations and functional food items based on analyzed chicory extracts could be developed, which may be applied, for example, for reducing Alzheimer’s disease symptoms. 

**Figure 3:**
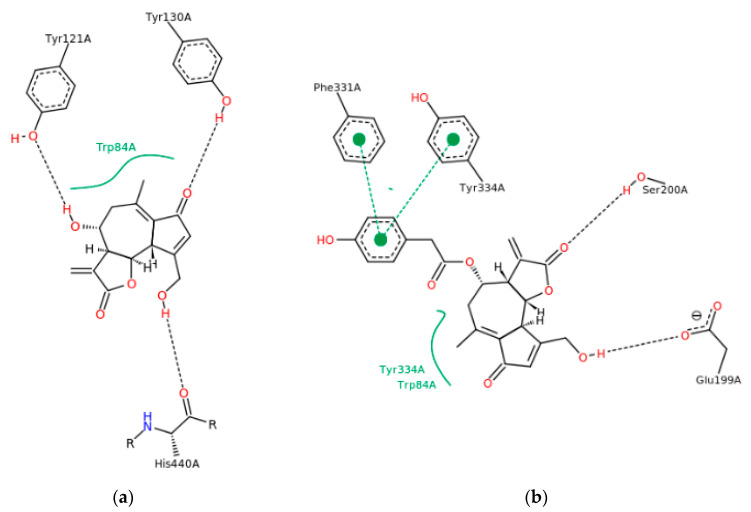

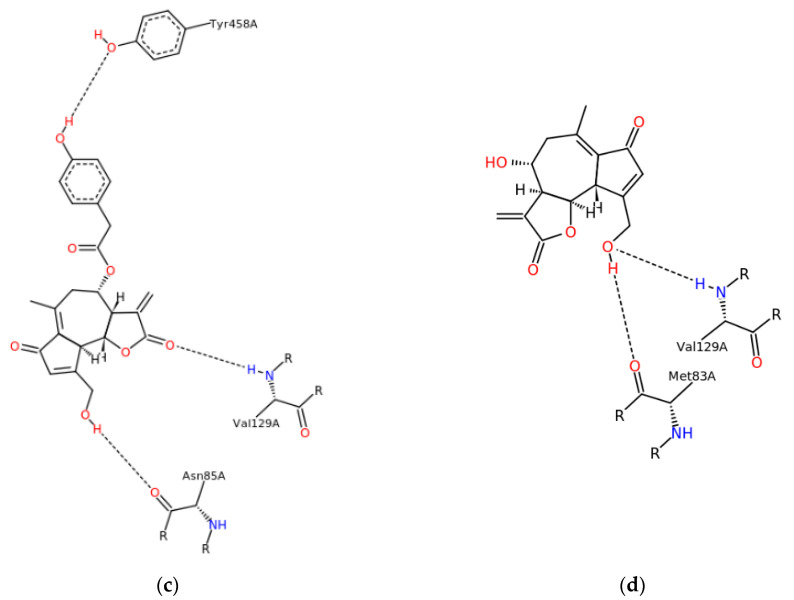
Depiction (in 2D) of the main interactions established between the active site of AChE and (**a**) lactucin; (**b**) lactucopicrin; (**c**) lactucopicrin in the presence of lactucin (the priority for docking was established for lactucopicrin); (**d**) lactucin in the presence of lactucopicrin (the priority for docking was established for lactucin). Continuous green lines represent hydrophobic interactions, while black dashed lines show hydrogen bonds and green dashed lines show pi-pi interactions.

**Figure 4:**
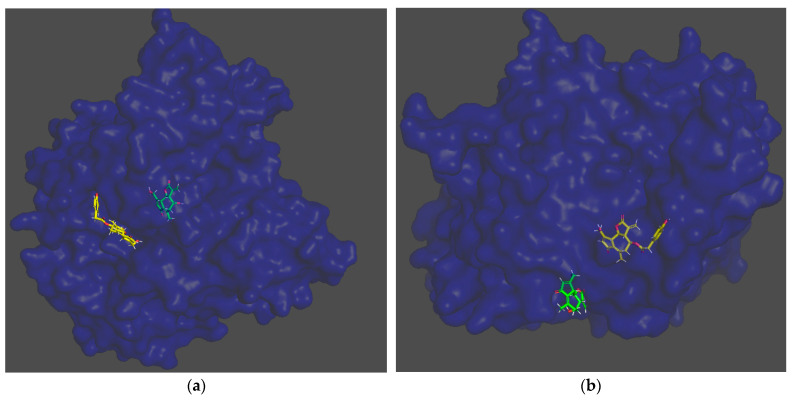
Docking simulation results. Three-dimensional models of positions in AChE pockets: (**a**) lactucin and lactucopicrin (the priority for docking was established for lactucin); (**b**) lactucopicrin and lactucin (the priority for docking was established for lactucopicrin). Chain markings: green—lactucin, yellow—lactucopicrin.

In the docking simulation, binding to the amino acid residues of the hydrolytic fold forming the active sites of AChE was considered [38]. In the case of lactucin, the docking simulation showed the presence of hydrophobic-type interactions and hydrogen bonds with TRP84, TYR121A, TYR130A and HIS440A, whereas lactucopicrin exhibited pi-pi type interactions with PHE331 and TYR334 as well as hydrophobic ones with TYR334 and TRP84A, accompanied by hydrogen bonds with PHE331, TYR334, SER200A and GLU199A. The type of interactions was different between the two substances and energy changes caused by complexation varied significantly (Figure 3a,b). The ΔG predicted by docking simulation amounted to −37.64 and −47.90 kJ/mol for lactucin and lactucopicrin, respectively, indicating lower enthalpy changes in complexation comparing to values calculated in ITC tests. The difference might come from the fact that in the ITC method, not only the binding at the active site is considered, but also possible interactions at other enzyme domains. Due to the fact that chicory extracts contain a mixture of lactones including lactucopicrin and lactucin, a docking simulation of both compounds in one calculation was also performed in two steps taking into account various docking priority variants (Figure 3c,d). In the 2D depiction of docking simulation of two substances, only the stronger interactions with one of the SLs are shown. In these approaches, only hydrogen bond formation was observed. Docking of the two inhibitors, taking into account the docking priority, revealed the energetic coupling between the ligands as well as the dynamics and intermediates of the two protein-binding ligands, which resulted in differences in bound active sites compared to docking one substance. They include MET83 and ASN85, which are adjacent to TRP84 at the anionic site of the peripheral active site, limiting the activity of this part of the enzyme pocket [39]. This observation proves that the docking simulation using more data leads to different results and, like the calorimetric titration, may be useful at the first step of screening the activity of complex extracts. Potential activity should then be validated in more complex in vitro and then in vivo models. In Figure 4, the 3D models of these interactions are shown. It could be observed that each of the SLs were bound to the enzyme at different part of the active site, preventing the enzyme from binding the substrate, which could be responsible for the better properties of the extracts than the single compound as an inhibitor of AChE hydrolytic activity.

Both sesquiterpene lactones and SL-containing chicory extracts showed AChE-inhibitory activity in ITC and docking simulation models. Several studies have shown the neuroprotective effect of lactucopicrin in cell lines, confirming the activity of SLs in more complex research models, including the one by Venkatesan et al. [40], where lactucopicrin increased intracellular Ca^2+^ levels causing a rise in muscarinic acetylcholine receptor expression in N2a cells and neurotrophins, including NGF, BDNF, and NT3 in C6 cells. Thus, other neuroprotective mechanisms, in addition to AChE inhibition, are also possible. 

Recent reports have described the activity of the known AD drugs: rivastigmine, donepezil and galantamine, which allows comparing the activity of natural substances. The study by Marucci et al. [41] showed a high inhibitory activity for AChE of donepezil IC_50_ = 5.7 nmol/L and calantamine IC_50_ = 11 nmol/L, while that of rivastigmine amounted to IC_50_ = 1.03 μmol/L and was at the level similar to the activity of the analyzed SLs. However, looking at the side effects of these drugs, the tested sesquiterpene lactones from chicory have high potential for further studies on AChE inhibition.

The research presented in this paper showed better properties of lactucopicrin in comparison with lactucin as an AChE-hydrolytic-activity inhibitor, which is consistent with the results of the cited authors, who emphasized the importance of the presence of lactucopicrin in chicory as the cholinesterase inhibitor. It could be observed in the results of the ITC analysis that the purified extracts were more effective than a single substance. The reason for this phenomenon may be the complementary binding of different SLs at several fragments of the enzyme active site.

**Figure 5:**
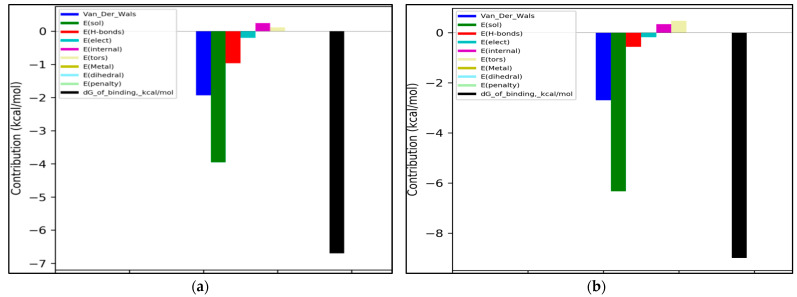
Energetic contributions (kcal/mol) to binding energy for ligand pairs’ docking: (**a**) lactucin in the presence of lactucopicrin (the priority for docking was established for lactucin); (**b**) lactucopicrin in the presence of lactucin (the priority for docking was established for lactucopicrin); Contributions (from left to right) depicted are: van der Waals interactions (navy blue), solvation energy (green), hydrogen bonds (red), hydrophobic interactions (light blue), repulsion (purple), energy of entropic losses associated with ligand’s rotatable bonds (yellow) and total predicted binding energy (black).

In Figure 5, the energetic contributions of different types of interactions to the total binding energy calculated during docking ligand pairs in two modes are shown. The most important type of interaction was the solvation energy which is the change in Gibbs energy during dissolving of the molecule, which could not be shown in 2D and 3D models. This type of interaction had higher energy for docking lactucin in the presence of lactucopicrin than in the reverse docking, although both graphs show similar energy distributions among specific types of interactions. Similarly, for the second important van der Waals interactions, a similar relation was observed. The hydrogen bonds and hydrophobic interactions in terms of the energy contribution had far lower significance. The forces responsible for the weakening of the complexes’ stability, such as the energy of entropic losses associated with the ligand’s rotatable bonds and repulsion, were at a relatively low level, which could be caused by a sufficient flexibility of the tested molecules. Further studies should take into account in vivo verification of the formation of SLs-AChE complexes in plasma, and their stability.

## 4. Conclusions

This study used isothermal titration calorimetry and docking simulation to provide relevant information at the molecular level on the AChE inhibition by two sesquiterpene lactones, lactucin and lactucopicrin, or chicory extracts, depending on the type of the solvent used and the further purification of extracts. 

The results showed that lactucopicrin might have more beneficial properties for AChE inhibition compared to lactucin. The chicory extracts containing significant (2–4%) concentrations of sesquiterpene lactones are potential preparations that could limit the in vivo hydrolysis of acetylcholine and should be consequently studied in more advanced models. They have great potential and, after further clinical trials, could be used to alleviate the symptoms of Alzheimer’s disease.

## Figures and Tables

**Table 1:** Concentration of sesquiterpene lactones from purified and unpurified extract *of Cichorium intybus* L.

Sesquiterpene Lactones (g/100 g db.)	Raw Extracts		Purified Extracts			
	70/30	50/50	CPC 70/30 I	CPC 70/30 II	CPC 50/50 I	CPC 0/50 II
8-Deoxylactucin	1.094 ± 0.084 ^a^	0.809 ± 0.068 ^b^	0.030 ± 0.004 ^d^	1.612 ± 0.034 ^c^	0.042 ± 0.009 ^d^	1.188 ± 0.044 ^a^
Lactucin	0.554 ± 0.038 ^c^	0.515 ± 0.041 ^c^	0.671 ± 0.023 ^a^	0.236 ± 0.021 ^b^	0.645 ± 0.016 ^a^	0.273 ± 0.014 ^d^
11(S),13-Dihydrolactucin	0.328 ± 0.025 ^b^	0.285 ± 0.017 ^d^	0.323 ± 0.011 ^b^	0.175 ± 0.023 ^a^	0.311 ± 0.001 ^b^	0.125 ± 0.021 ^c^
8-Deoxylactucin oxalate	1.129 ± 0.074 ^c^	0.946 ± 0.075 ^b^	0.100 ± 0.018 ^a^	1.830 ± 0.014 ^e^	0.105 ± 0.011 ^a^	1.359 ± 0.033 ^d^
Lactucopicrin	1.291 ± 0.054 ^a^	0.045 ± 0.003 ^d^	1.303 ± 0.001 ^e^	0.418 ± 0.012 ^c^	0.085 ± 0.028 ^b^	0.004 ± 0.001 ^f^
11(Z),13-Dihydrolactucopicrin	0.083 ± 0.002 ^b^	0.057 ± 0.008 ^c^	0.120 ± 0.021 ^d^	0.007 ± 0.009 ^a^	0.088 ± 0.014 ^b^	0.006 ± 0.001 ^a^
Total sesquiterpene lactones	4.479 ± 0.297 ^a^	3.457 ± 0.212 ^d^	4.267 ± 0.013 ^f^	4.278 ± 0.021 ^c^	2.041 ± 0.005 ^e^	2.955 ± 0.014 ^b^

70/30, water–methanol (70/30, *v*/*v*) extract; 50/50, water–methanol (50/50, *v*/*v*) extract; CPC (countercurrent partition chromatography) —extracts after purification by countercurrent partition chromatography collected in two fractions, in time intervals: I—from 8 to 13 min and II—from 14 to 18 min; the same superscript letter in one row indicates no statistically significant differences between extracts (*p* < 0.05).

**Table 2:** Thermodynamic parameters of the interaction of chicory extracts with AChE based on the ITC analysis.

Substance/ Extract	∆H [kJ/mol]	∆G [kJ/mol]	ΔGp [kJ/mol]	∆S [J/mol × K]	K_D_ [µmol/L]	K_A_ × 10^3^ [L/mol]	IA [%]	IC_50_ [μmol/L]	K_I_ [µmol/L]
Lactucin	−166.93 ± 0.22 ^e^	−23.41 ± 1.82 ^a^	−37.64	−262.62 ± 6.21 ^b^	29.90 ± 0.11 ^f^	33.44 ± 0.21 ^a^	26.23 ± 0.12 ^e^	3.11 ± 0.21 ^a^	1.12 ± 0.06 ^c^
Lactucopicrin	−74.45 ± 0.88 ^d^	−22.41 ± 1.59 ^a^	−47.90	−262.27 ± 2.21 ^b^	169.00 ± 0.12 ^c^	5.92 ± 0.11 ^d^	79.07 ± 1.09 ^d^	1.74 ± 0.11 ^b^	1.32 ± 0.11 ^b^
CPC 70/30 I	−69.93 ± 0.76 ^c^	−20.94 ± 1.13 ^a^	-	−212.14 ± 9.87 ^a^	147.00 ± 0.99 ^d^	6.81 ± 0.11 ^c^	91.19 ± 2.15 ^c^	1.65 ± 0.13 ^b^	1.16 ± 0.06 ^a^
CPC 70/30 II	−58.74 ± 0.54 ^b^	−22.24 ± 1.58 ^a^	-	−263.68 ± 12.87 ^b^	299.02 ± 0.87 ^a^	3.34 ± 0.04 ^f^	98.41 ± 1.22 ^b^	1.55 ± 0.16 ^b^	1.18 ± 0.04 ^a^
CPC 50/50 I	−58.49 ± 0.33 ^b^	−22.99 ± 2.09 ^a^	-	−289.61 ± 10.81 ^c^	180.01 ± 0.66 ^b^	5.56 ± 0.16 ^e^	98.61 ± 0.14 ^a^	1.51 ± 0.32 ^b^	1.43 ± 0.11 ^b^
CPC 50/50 II	−57.72 ± 0.16 ^a^	−23.43 ± 1.82 ^a^	-	−261.92 ± 11.09 ^b^	134.02 ± 0.87 ^e^	7.46 ± 0.21 ^b^	98.26 ± 0.12 ^b^	1.53 ± 0.21 ^b^	1.12 ± 0.13 ^a^

70/30, water–methanol (70/30, *v*/*v*) extract; 50/50, water–methanol (50/50, *v*/*v*) extract; CPC—extracts after purification by countercurrent partition chromatography collected in two fractions, in time intervals: I—from 8 to 13 min and II—from 14 to 18 min; ΔG—affinity of the inhibitor; ΔGp—affinity of the inhibitor predicted using docking simulation; K_D_—dissociation constant; K_A_—binding constant; IA—inhibition activity; K_i_—inhibition constant; n = 9, ± SD, the same superscript letters in one column indicate no statistically significant differences between the extracts *p* < 0.05.

## Data Availability

Data available on request due to restrictions, e.g., privacy or ethical. The data presented in this study are available on request from the corresponding author. The data are not publicly available because they are part of the authors’ own research.
